# Data Mining for Gene Networks Relevant to Poor Prognosis in Lung Cancer Via Backward-Chaining Rule Induction

**Published:** 2007-02-10

**Authors:** Mary E. Edgerton, Douglas H. Fisher, Lianhong Tang, Lewis J. Frey, Zhihua Chen

**Affiliations:** 1 Department of Pathology and Department of Biomedical Informatics, Vanderbilt University, currently Department of Anatomic Pathology, University of Texas, M.D. Anderson Cancer Center, 1515 Holcombe Boulevard, Houston, TX 77030; 2 Department of Electrical Engineering and Computer Science, Vanderbilt University; 3 Vanderbilt-Ingram Cancer Center, currently Department of Biomedical Informatics, Vanderbilt University; 4 Department of Biomedical Informatics, Vanderbilt University, currently Department of Biomedical Informatics, University of Utah School of Medicine, Salt Lake City, UT 84112; 5 Department of Electrical Engineering and Computer Science, Vanderbilt University, currently Department of Interdisciplinary Oncology, H. Lee Moffitt Cancer Center and Research Institute, SRB3, 12902 Magnolia Drive, Tampa, FL 33612

**Keywords:** microarray, data analysis, molecular mechanisms, class discovery, semi-supervised methods, decision trees, C4.5, non-small cell lung cancer, systems biology

## Abstract

We use Backward Chaining Rule Induction (BCRI), a novel data mining method for hypothesizing causative mechanisms, to mine lung cancer gene expression array data for mechanisms that could impact survival. Initially, a supervised learning system is used to generate a prediction model in the form of “IF <conditions> THEN <outcome>” style rules. Next, each antecedent (i.e. an IF condition) of a previously discovered rule becomes the outcome class for subsequent application of supervised rule induction. This step is repeated until a termination condition is satisfied. “Chains” of rules are created by working backward from an initial condition (e.g. survival status). Through this iterative process of “backward chaining,” BCRI searches for rules that describe plausible gene interactions for subsequent validation. Thus, BCRI is a semi-supervised approach that constrains the search through the vast space of plausible causal mechanisms by using a top-level outcome to kick-start the process. We demonstrate the general BCRI task sequence, how to implement it, the validation process, and how BCRI-rules discovered from lung cancer microarray data can be combined with prior knowledge to generate hypotheses about functional genomics.

## Introduction

Development of therapies to improve outcomes in lung cancer, the leading cause of death from cancer in this country, lags behind other cancers. Recent developments in therapies that have been designed to interfere with specific molecular targets, such as gefitinib, an inhibitor of tyrosine kinases activated by the epidermal growth factor receptor (EGFR), or the p53 vaccine, have not had the impact on survival that was hoped for. A major problem for the success of a molecularly targeted therapy is the lack of knowledge about the myriad of gene interactions that lead to resistance. In fact, a recent publication (Ogino et al. 2005) describes evidence for the interactions of CDKN1A/p21 and p53 in conferring resistance to chemotherapy combined with gefitinib in colon cancer patients. This discovery and others like it point to the need to identify the coupling of gene networks relevant to cancer progression. Gene expression array data, by virtue of its simultaneous measure of the expression of thousands of genes, has the potential to deliver the information that can identify the gene interactions that act to link active network.

Data analysis tools for microarray data have largely focused on methods to accurately classify an outcome with validation on a separate test cohort. Less attention has been given to identifying the gene interactions that operate within a patient class as defined by outcome. It is clear that the set of all combinations of all possible gene interactions would be too large to explore. Our task is to devise a computer strategy that can explore a very large space of gene interactions, enriched for plausibility, and reduce them to a manageable set for human consideration (e.g. Huels et al. 2002).

We stress here that accurate prediction of an outcome does not necessarily include information concerning the gene interactions that can occur in an aggressive tumor. These interactions may be only loosely tied, in a statistical sense, to the outcome. However, they can be very important in moderating the activity of a critical gene, or in conferring resistance to therapy once a critical gene target is removed. We illustrate here the use of Backward Chaining Rule Induction, recently described in a publication by Fisher et al. (2005), to address this paradigm and we discuss validation methods for this type of analysis. BCRI extends prior research into exploratory strategies (Evans and Fisher, 1994; Evans and Fisher, 2002; Waitman et al. 2003; Waitman et al. in press) based on rule induction. The analysis in this paper significantly extends Fisher et al. (2005), where we first introduced BCRI to the computer science and data mining community as a strategy for mining gene expression array data.

Our approach is focused on *hypothesis generation* (and data mining generally), as opposed to hypothesis testing and classification. Both the methodology of the data mining and the means of validating the results are different for pathway discovery relevant to a clinical outcome versus classification of a clinical outcome. Backward Chaining Rule Induction as a strategy differs from the use of rule induction, or any method for that matter, to building a classifier to predict an outcome. Classifiers are validated by evaluating their accuracy in predicting outcomes on a test set of patients. Gene interactions are validated against existing knowledge in pathways databases and published literature, or presented as hypotheses for further study at a basic science level.

Following our Introduction here (Section 1), we describe the Data and our rationale behind creating two survival classes in Section 2. We use published gene expression microarray data generated by Beer et al. (2002) as our source of microarray data. We describe the BCRI strategy and outline its implementation in this illustration in our Methods Section (Section 3). Finally, we survey two comprehensive pathways databases along with published literature for validation of the gene interactions discovered from the results generated by BCRI (Section 4). Of course, novel interactions learned from BCRI may not yet exist within these databases and we discuss the use of BCRI to generate hypotheses about these new interactions. In our Conclusions, (section 5) we discuss future work with BCRI that extend our ideas to the use of automating knowledge assessment for validation of the interactions learned from BCRI.

## Data

We established two criteria for our selection of published gene expression microarray data in this illustration. Our criteria for the selection was that the data (1) be well characterized and (2) have already proven itself as a resource for identifying statistically significant molecular correlates predictive of lung cancer outcome.

In 2002, Beer et al. (2002) generated a set of gene expression profiles for 86 patients with resectable lung adenocarcinoma. All patients underwent surgery, did not receive neo-adjuvant therapy, and their survival intervals from the time of diagnosis were reported. Molecular correlates identified by Beer and co-workers were used to separate statistically significant differences in survival data using microarray data generated by a separate cohort (Bhattacharjee et al. 2001). Using the same data, a classifier for nodal involvement was found to be accurate in predicting node positive patients but not node negative patients. Interestingly, the node negative patients that were misclassified had a statistically significant worse outcome, suggesting the possibility of occult metastases (Xi et al. 2005). Thus, this data met our criteria.

We used the data as processed and normalized by the original authors. They determined transcript abundance using a customized algorithm. They filtered the data to exclude genes if the measure of their 75th percentile value was less than 100. Their final set consisted of 4996 genes. We used this expression set along with eleven clinical attributes that they published. The clinical attributes were age, gender, T and N status (as described above), stage of disease as per the current American Joint Commission on Cancer specified algorithm based on T, N, and M, histopathological subtype of adenocarcinoma, histopathological grade of the tumor, smoking history, survival, p53 mutation status, and K-ras mutation status. Overall, the vast majority of attributes are continuous with very few nominal attributes.

### Optimal split point determination

We divided the patients into a high risk group that died within 30.1 months of diagnosis, and a low-risk group that lived beyond this time interval. The BCRI strategy itself is independent of the method used to determine the optimal split point to divide the high and low risk survival classes. But because backward-chaining is initiated using the classes defined by the split point, the relevance of the BCRI results is expected to be a function of how accurate the prediction model is at that split point (i.e. how well that split point corresponds to different underlying disease mechanisms). Therefore, we completed an exhaustive study of the leave-one-out-cross-validation prediction error calculated at all possible split points. The approach to such a study and the analyses required to insure against a false discovery or the bias introduced by the ratio of class membership has been addressed in detail in our laboratory. We briefly describe it here.

**Figure 1.**
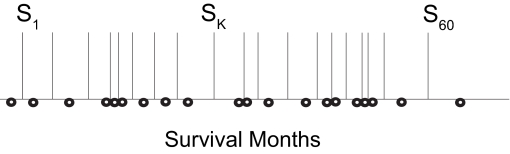
candidate split points.

Figure 1 below is used to illustrate the procedure of testing split points for the two survival classes (note that each of the 60 split points in not actually depicted in this schematic). Each open circle in Figure 1 represents the survival time of a patient in months, and these are sorted into ascending order. The median between each two adjacent open circles is a candidate split point, and is designated by a vertical line. For the 61 records retained in our study, we considered 60 candidate split points. We label three of these lines in Figure 1 for purposes of later illustration as S_1_, the first candidate split point, S_k_, the k-th candidate split point, and S_60_, the 60th or final candidate split point.

Again, our task was to select the “best” split point for distinguishing high and low risk patients. Thus, for each candidate split point,

1. We divided the data into two sets, one with survival time less than (to the left of) the split point, and one set with survival time greater than (to the right of) the split point. The data to the left was labeled “high risk” and the data to the right was labeled “low risk.”
2. We determined the LOOCV prediction error rate using the C4.5 system for learning decision trees. (We describe the C4.5 algorithm in more detail in the Methods Section and in Appendix A,) Intuitively, we assume that the split point that separates the data into biologically meaningful groups will lead to ideal predictive performance. It would be tempting to use that split point for which C4.5 LOOCV error rate was minimized, but we insured that the minimum in the predicted error was not simply a function of the bias introduced by the proportional sizes of the classes at that particular split point. This is an important consideration because in the extreme, a split point that placed 60/61 samples in one class and 1/61 (e.g. S1 or S60) in another class would lead to what appeared to be a highly accurate classifier using even the most trivial prediction rule (e.g. predict the most common class). However, such a split point would have no biological relevance. Thus, for each candidate split point,
3. We computed the prediction error obtained if we simply predicted the most common class. Rather than selecting the split point that minimized C4.5 LOOCV error rate, we selected that split point for which the C4.5 LOOCV error rate was maximally less than the most-common-class error rate for that split point. This occurred at 30.1 months, which had a C4.5 LOOCV error rate of 8.2% and a most-common class error rate of approximately 35%.

### False discovery rate

As further evidence that this split results in meaningful class distinctions, we estimated the *false discovery rate* at the 30.1 month split point. In particular, we created classes of 19 and 42 members (i.e. the proportions of High and Low risk patients, respectively, with the 30.1 split point), by randomly drawing patients from the total set without replacement and assigning them arbitrarily to “high” and “low” risk classes. We applied C4.5 to an using a LOOCV design to discriminate these arbitrary classes. We performed this experiment 20 times. The mean error across these 20 trials was approximately 40%. The lowest error rate among the 20 trials was approximately 24%, well above the true-data C4.5 LOOCV error rate of 8.2%.

### Data summary

In summary, our final selection of data consisted of 19 (31%) high risk patients and 42 (69%) low-risk patients. The total number of patients was 61. Forty-eight (78%) patients had Stage I disease and thirteen (22%) patients had Stage III disease at diagnosis. Of the patients with Stage I disease, eight were high risk and 40 were low risk. Of the Stage III patients, two were low risk and eleven were high risk. The LOOCV prediction error with the 30.1 month split point was 8.2% using the C4.5 decision tree algorithm to build prediction models. As stated above, this summary is expanded in a technical paper that is in preparation, and this result has limited relevance to the current discussion: if we identify a split point that minimizes error and possibly corresponds to different disease mechanisms, then the more likely the relevance and interestingness of genomic interactions suggested by BCRI.

## Methods

Rule induction algorithms have been used in multiple studies to analyze gene expression array data. Virtually all of these studies have used supervised rule induction (e.g. Cho et al. 2001). However, since supervised approaches exclude rules that do not directly predict the outcome of interest, they omit mechanisms that are loosely tied to an outcome, but are relevant in terms of the potential to confer resistance. Given that the Knudsen “two-hit” model of cancer describes a sequence of events, the later ones modifying the former, we can expect that some very relevant mechanisms of interest will be common to all outcomes, and therefore would not be discovered using a supervised approach.

On the other hand, the problem with using purely unsupervised rule induction to hypothesize gene interactions and networks is that the space of all possible gene interactions that can occur is too vast to effectively search. The resulting set may not be manageable for examination by experts and assignment of clinical relevance, or discovered rules may not be clinically relevant at all. To be practical, the search for plausible gene interactions must be focused.

Our goal is to embed rule induction in a semi-supervised approach. We have introduced the strategy of Backward Chaining Rule Induction (BCRI) to that effect (Fisher et al. 2005). BCRI initially applies a rule induction algorithm to discover the conditions that predict a clinical outcome—*long* versus *short* survival periods for lung cancer patients in the case of the research reported here. This produces a set of human readable rules of the form “IF <conditions> THEN <outcome>,” as described above. This type of rule, which implies an interaction, is of particular interest because it can be translated into hypothetical mechanisms for subsequent validation. For example, the rule

IF < (A-kinase anchoring protein expression is greater than 496) AND (urea transporter expression is less than or equal to 397) >

THEN < Annexin V, a phospholipase inhibitor, expression levels are greater than 750 >.

can be translated into a hypothesis stated as up-regulation of A-kinase in combination with down-regulation of the urea transporter causes the phospholipase inhibitor Annexin V to be down regulated.^1^

In BCRI, rule induction is used recursively to generate a prediction model for the antecedents (IF conditions) of rules from the previous model. This backward chaining step is repeated, treating antecedents from earlier steps as outcomes until a termination condition is satisfied (e.g. the number of samples remaining to classify or the accuracy of the classifier is below a user defined minimum). The intent of backward-chaining rule induction is to better focus the search (than it is through strictly unsupervised means) for causal mechanisms by using outcome (e.g. survival) to kick-start the process. Thus BCRI is intermediate between supervised rule induction and unsupervised rule induction. Rather than a relatively unconstrained exploration of the space of associations between variables, as would occur in unsupervised rule learning, only the associations that are traceable to a top-level class are examined.

Antecedent conditions found in the rules that predict outcome then become “sub-goals,” and rule induction is repeated on the data using these sub-goals as classes. The process of backward-chaining on rule antecedent conditions is repeated until a termination condition is satisfied.

Once we have generated a set of rules, we analyze these for AND based rules that imply a potential interaction between two genes. We also examine the relationship between the gene(s) specified in the antecedent condition and the consequent. The OR conditions would be considered to be independent pathways that results in the same consequent. In this introduction of BCRI, we use well characterized interactions learned from PubMed and GeneCards along with chromosomal location learned from LocusLink as established domain knowledge with which to compare the interactions hypothesized from BCRI. A more detailed projection of the interactions hypothesized by BCRI superimposed on known pathways using MetaCore™ (Nikolsky et al. 2005) and Pathway Assist™ (Nikitin et al. 2003) is discussed in the context of validation and generating new hypotheses.

### C45W-BCRI: An implementation of BCRI

We distinguish the general BCRI strategy from the possible implementations of this strategy. This paper describes the general BCRI strategy as well as a particular implementation of that strategy which we call C45W-BCRI. Other possible implementations are briefly discussed at the end of the paper. BCRI has also been described in a recent publication by our group (Fisher et al. 2005).

Our initial approach implements BCRI as a “wrapper” around a rule-induction engine This design, while not the most efficient from a computational point of view, makes sense in early system development because it allows us to plug in and experiment with different rule-induction algorithms. We anticipate that in the future, at a later stage of BCRI’s evolution, more tightly coupling the rule induction algorithm with other components of BCRI will lead to more efficient implementations.

In the wrapper-based prototype, BCRI is passed the labeled gene expression data, a set of the target classes used to label the data, and three functions: ***RuleInducer, PriorityFn, and TerminateFn.*** We have included pseudocode (the C code with local variable declarations excluded) in Table 1 for those interested in a technical description of the code.

***RuleInducer*** is used to designate the rule induction engine that generate the rules to predict the classes that were passed to it, called *Target-Conditions*. ***PriorityFn*** creates a priority queue of rules generated in ***RuleInducer*** that should be used to generate the next set of *TargetConditions*. The loop is terminated by ***TerminateFn***, which uses a condition, in this case the rule depth, to determine whether to continue to create new classes (*TargetConditions*) to pass to ***RuleInducer*** or stop.

***RuleInducer*** can, in principle, be any supervised rule discovery system that, given a class, will return rules that predict that class. Our current implementation adapts a well-known system, C4.5 (release 8), developed by Quinlan (1993) for ***RuleInducer***. We point out some of the implications of this choice and other possible options for rule induction engines in the Discussion Section.

C4.5 is a supervised method for learning decision trees, from which if-then rules of the type already illustrated can be extracted. One of our motivations in using C4.5 for our first implementation of BCRI is because of its inherent ability to utilize both discrete and continuous variables. The algorithm is well known and we refer the interested reader to Quinlan’s textbook for a more detailed description. In brief, C4.5 is a recursive, greedy algorithm for building a decision tree from the top down. Starting with the empty decision tree and the set of training data, C4.5 first selects the attribute that “best” predicts class with respect to the training data. It measures the decrease in the information-theoretic measure of entropy (i.e. information gain ratio) as the basis, of this selection. C4.5 continues to recursively examine the ability of every attribute to partition the remaining data until there are no data left or until the attribute value selected to partition the data covers an insufficient number of samples in the training set.

Using an information theoretic measure to assess continuous attributes requires special consideration. C4.5 sorts the values of any continuous variable, and considers the median between two consecutive values as a possible threshold to separate continuous variables into two values.

As described in Section 2. on Data, we first create a binary scheme of classification based upon survival above or below a certain threshold. This is the “zero-th” level *TargetCondition*. In ***RuleInducer*** we employ C4.5 (release 8) to output a set of if-then rules that predict *TargetCondition* using its default settings. We wrote a small portion of code to parse the C4.5 output and convert the decision trees into if-then rules with associated coverage (i.e. the number of instances in the data that satisfy the rule’s antecedent) of the rule for subsequent analysis by ***PriorityFn***. We use the abbreviation COV to designate coverage in our presentation of the results in the next section.

***PriorityFn*** is applied to a rule and returns a score. This score is used to store the rule on a priority queue of other scored rules. In our current implementation, the coverage (COV) of the rule, is used to organize the priority queue. Other possibilities include the rule’s accuracy or the like. Our choice of coverage, versus accuracy or a like measure, is motivated by the observation that rule-learning systems will tend to produce accurate rules (relative to a data specific upper bound), but that these rules will vary significantly in coverage. We prefer to favor rules that cover a large proportion of data.

At each iteration of ***RuleInducer***, a new binary scheme for *TargetCondition* is defined based upon the rules. A decision tree is generated to predict these *TargetConditions*. In the case of discrete attributes in rules, we consider the next generation Class 1 as the instances for which the data satisfies the rule and Class 2 as all other instances for which the data is not covered by that rule. For example, Stage I is the root level attribute selected to classify patients into high and low risk survival classes. In the next iteration, the *TargetConditions* are the Stage I patients as one class and all of the patients who are not Stage I as the other class. If the antecedent condition of a rule is based upon a continuous threshold, then that threshold value is used to divide the data into two classes. In the case of an antecedent rule that involves the conjunction of two conditions, then each condition is considered separately in the next iteration (and prioritized by its coverage). Consider the antecedent condition for the rule given as an example earlier in this section

IF < (A-kinase anchoring protein expression is greater than 496) AND (urea transporter expression is less than or equal to 397)>

There would be a set of *TargetConditions* defined according to whether or not the A-kinase anchoring protein expression value was greater than 496 or not, and a separate set of *TargetConditions* defined according to whether the urea transporter expression was above the value of 397 or not. Each of these two separate sets of binary *TargetConditions* would be assigned a priority depending upon their coverage, and separately submitted to ***RuleInducer*** to generate a decision tree at the next depth.

We continue the iteration until ***TerminateFn***, which indicates whether a rule should be further expanded (ie, continued backward chaining on its antecedents), returns a value of false. In this implementation we use a depth bound of three to terminate BCRI (with the first rule to predict high and low risk for survival classes as the zero-th level class). Other strategies that could be considered would include specifying a minimal coverage or confidence bound.

## Results

**Figure 2.**
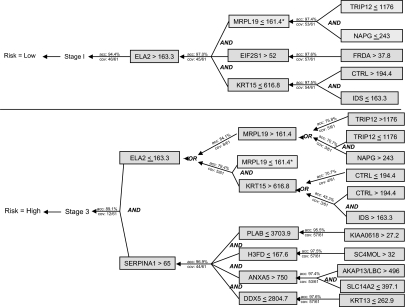
C45W-BCRI rules expressed as an AND/OR graph. *The rule to predict MRPL19 < 161.4 in the Low Risk trace will also predict MRPL19 < 161.4 in the High Risk trace. It is not shown again in the High Risk trace.

C45W-BCRI is our wrapper-based implementation of BCRI with C4.5 playing the role of ***RuleInducer***, and with ***PriorityFn*** and ***TerminateFn*** as specified immediately above. All rules given in this paper were produced by C45W-BCRI. The definitions for the gene abbreviations used in these rules generated by C45W-BCRI are in Table 2. Rule Depth is defined as the iteration number used to generate the rule. For example, the top most rule for the classifier for outcomes is depth zero (0). In Figure 2, depth zero is Stage I for low risk patients and stage III (or 3 as it is depicted in the figure) for high risk patients. The first set of rules that predict Stage are depth one. BCRI was terminated at a rule depth of three.

Except where noted, the abbreviations in Table 2 are accepted as HUGO nomenclature. Where noted by an asterisk, we have used common names supplied from Affymetrix™ for the relevant probe, and we have added the HUGO approved nomenclature in parentheses. We will discuss their function and potential role in lung cancer in our Discussion section, following a brief description here of an example trace of the BCRI procedure.

### Application of C45W-BCRI in the domain of lung cancer

Using High and Low Risk as the top-level classification, C45W-BCRI begins with (Risk = Low) (cov: 42/61) and (Risk = High) (cov: 19/61) placed on the priority queue (i.e. passed as Classes to C45W-BCRI). The term “cov” is an abbreviation for data coverage, or the number of patients out of the total number of patients that belong to the specific class (described above in ***RuleInducer***) for the condition just described.

(Risk = Low) is dequeued. Application of ***RuleInducer*** in the C45W-BCRI implementation yields a single rule, which is placed on the queue:

[(Stage = 1) → (Risk = Low) (cov: 48/61) ‖ (Risk = High) → (cov: 19/61)].

(Stage = 1) → (Risk = Low) is dequeued and ***RuleInducer*** yields a rule, which is added to the queue:

[(ELA2 > 163.3) → (Stage 1) (cov: 46/61) ‖ (Risk = High → (cov: 19/61) ]

The first of these rules is dequeued. A new rule is learned:

(MRPL19<= 161.4) & (EIF2S1 > 52) & (KRT15 <= 616.8) → (ELA2 >163.3) (cov: 45/61)

This rule is queued, resulting in the following priority queue:

[(MRPL19<= 161.4) & … → (ELA2 >163.3) (cov: 45/61) ‖ (Risk = High → (cov: 19/61)]

Having the highest priority (coverage), this same rule is immediately dequeued. Each individual antecedent serves in turn as a class and rules. A simple depth bound of three is used to terminate backward chaining, and these labeled rules are terminal.

Table 3 shows the 19 rules learned from the lung cancer data by backward chaining to depth of three, beginning with an initial queue of

[(Risk = Low) → ‖ (Risk = High) → ]

Gene definitions have been given already in Table 2. Coverage (“cov”) has already been defined; “acc” denotes accuracy, which is obtained using the error estimate for each node that is calculated by C4.5. As Figure 2 illustrates, the network of rules learned by BCRI is an AND/OR graph, much like the rule bases of expert systems such as Mycin (Shortliffe et al. 1975). We discuss the inference possibilities of such networks in the **Summary** section at the end of the paper. Our primary goal here is a limited, focused exploration of the associations between variables, which is directly (initially) or indirectly (as backward chaining proceeds) tied to top-level class(es).

## Discussion

### Gene interactions learned from BCRI: cancer and lung cancer relevance

Our hypothesis is that BCRI will generate a set of rules that are densely populated by “interesting” rules when initiated by top-level classes of interest. We expect BCRI to generate interactions that are already well-known, interactions that are partially supported, and novel interactions that can be used to generate hypotheses about networks relevant to lung cancer. We have evaluated the C45W-BCRI rules using PubMED, LocusLink (Pruitt et al. 2000; Pruitt and Maglott, 2001), and GeneCards (Rebhan et al. 1997) as sources for peer-reviewed literature, chromosomal location, and functional annotation, respectively.

Excluding the selection of Stage in the two topmost level rules, 17 rules were generated with 19 molecular species. Of the 19 molecular species selected in the rules, 12 have been associated with neoplasia in general and five specifically with lung cancer (see Table 4 and discussion below). Using MetaCore™ (Nikolsky et al. 2005), a pathway database tool, we find that ten of the species are connected by known pathways within a distance of 3 nodes. Of the 17 rules with gene interactions discovered in the C45W-BCRI session, 12 are evaluated as plausible in terms of our knowledge base today. Of these, six of the interactions have been specified previously in the literature related to lung cancer, and six are new association with lung cancer.

The evaluation for the inferences made from the rules is discussed below and summarized in Table 5. The interesting rules with plausible associations are labeled (*i*) in Table 3. The rule discussion and evaluation is based on domain knowledge from PubMed, LocusLink and GeneCards. The label was based on whether the interaction had been reported in the literature, if any of the genes involved has been associated with cancer or lung cancer; or if the gene loci have a close spatial relationship.

### ELA2 and SERPINA1

The first rules that we will examine are those that predict the top most level, Stage I and Stage III. In these rules we find a role for elastase in low risk tumors, and for its association with alpha-1-antitrypsin (SERPINA1) in high risk tumors. Within the rules Stage, although usually expressed in Roman numerals, will be assigned Arabic numbers for simplicity.

Rule # 2./Depth 1

(ELA2 > 163.3) → (Stage = 1)

[acc: 94.4% cov: 46/61]

Rule # 8./Depth 1

(ELA2 ≤= 163.3) & (SERPINA1 > 65) → (Stage = 3)

[acc: 89.1% cov: 12/61]

The association of the gene products for these two transcripts is well known as alpha-1-antitrypsin (SERPINA1) inhibits elastase (ELA2). An imbalance in activity between these enzymes in favor of elastase has been considered to play a role in the development of emphysema in smokers and in patients with alpha-1-antitrypsin deficiency. The source of elastase in the etiology of emphysema is from neutrophils and not from “native” lung cells. Elastase is synthesized during neutrophil development in the bone marrow, and packaged for release at sites of cellular injury. Given that the half-life of mRNA is orders of magnitude less than that for a neutrophil, the source of elastase transcript cannot be attributed to neutrophils in this data.

There have been a number of publications over the last decade suggesting a role for both elastase and alpha-1-antitrypsin separately in lung cancer. There have been minimal or no experiments focused on their association as it relates to lung cancer. Thus, the association discovered here is all the more interesting since both gene products have been implicated separately in lung cancer research. The next step is to identify whether a plausible model based on the association is compatible with previously published results. The publications have been sufficiently sparse that a putative role in cancer is not described in the GeneCards reference for either the elastase or alpha-1-antitrypsin gene.

Elastase has been proposed as a tumor promotor based on constitutive production of elastase in animal models of lung cancer and in lung cancer cell lines (Kamohara et al. 1997; Inada et al. 1997; Inada et al. 1998; Doi et al. 2002). High levels of elastase protein in lung tumor tissue have been correlated with higher stage tumors and poor survival in patients with lung cancer (Yamashita et al. 1996; Yamashita et al. 1997). In an epidemiological study, Taniguchi et al. (2002) demonstrated that single nucleotide polymorphisms in the promotor region for elastase are associated with a higher relative risk of having lung cancer.

In a 1992 study of adenocarcinomas, high levels of alpha-1-antitrypsin protein product were also found to be associated with higher stage disease (Higashiyama et al. 1992). Rule # 8, learned from C45W-BCRI, predicts that higher stage lung cancer results from lower levels of elastase transcript, and might at first appear to contradict the results published by Yamashita et al. (1997). However, the decrease in elastase *transcript* in our rule may be secondary to negative feedback in the setting of a high concentration of elastase *product*. The increase in transcribed alpha-1-antitrypsin may also be in response to the elevated elastase product or activity. As a result of this rule discovery, increased elastase activity or increased survival of the translated protein could be hypothesized as a mechanism for the higher levels of both active elastase product and of alpha-1-antitrypsin associated with Stage III lung cancer, and with poor survival. It has been postulated that increased elastase and similar proteinases destroy the barrier between tumor and the local circulatory system, either lymphatic or hematogenous, and result in at least loco-regional metastases. The increase in alpha-1-antitrypsin may have a role in promoting aggressive behavior in addition to that of elastase, or it may simply correlate with aggressive tumor behavior because of its up-regulation secondary to elastase activity.

Using the same logic for transcript production, Stage 1 tumors may have low levels of active elastase. If removal of elastase by normal cell mechanisms is sufficiently efficient, then disruption of local barriers to circulation may not occur. There may as yet be a mechanism by which increased transcription of elastase promotes tumor formation but not tumor progression, as suggested by the animal models described above.

In a recent review of the roles of elastase in lung cancer, Sun and Yang (2004) posed the question of “why there are more neutrophils or released neutrophil elastase in aggressive or late-stage tumours compared with less aggressive or early stage tumours?” They also pointed to the fact that the interplay between elastase and alpha-1-antitrypsin, rather than each one individually, needs to be addressed. Based on our data analysis, we would modify their question and ask *why is increased elastase production by lung tumor cells associated with tumorigenesis at all?* And *what is the role of alpha-1-antitrypsin if any?*

Here, C45W-BCRI supports a previously hypothesized role for elastase in lung cancer, and is compatible with previously reported data for both elastase and for aplpha-1-antitrypsin. C45W-BCRI discovers rules that are used to construct a unifying model that explains several separate publications on both elastase and alpha-1-antitrypsin.

### MRPL19, EIF2S1, and KRT15

In the next level of rules learned from C45W-BCRI, we find that the mitochondrial precursor of 60S ribosomal protein L19 (MRPL19) predicts decreased elastase transcription. It associates with eukaryotic translation initiation factor 2 subunit 1 (EIF2S1) and cytoskeletal keratin (KRT15) to predict high levels of elastase transcript. Either higher transcription levels of MRPL19 predict low levels of transcription of ELA2 in the “high risk” lineage of the model,

Rule # 3./Depth 2

(MRPL19 ≤ 161.4) & (EIF2S1 > 52) & (KRT15 ≤ 616.8) → (ELA2 > 163.3)

[acc 97.0% cov: 45/61]

Rule # 9./Depth 2

(MRPL19 > 161.4) → (ELA2 ≤ 163.3)

[acc: 84.1% cov: 8/61]

Rule # 12./Depth 2

(MRPL19 ≤ 161.4) & (KRT15 > 616.8) → (ELA2 ≤ 163.3)

[acc: 79.4% cov: 5/61]

Of these genes and their products, the 60S ribosomal protein L19, mitochondrial precursor (MRPL19) is the only one that lacks any references associated with any neoplastic behavior. Increased expression of eukaryotic transcription factor 2 subunit 1 (EIF2S1 or EIF2A1), a member of the complex required to translate from transcript to protein, has been associated with several tumor types, with c-myc, an oncogene important in lung tumorigenesis, and with specific subtypes of lung cancer (e.g. Rosenwald et al. 1993; Lobo et al. 2000; Koessler et al. 2003; Clemens, 2004; Rosenwald, 2004). Its activity is modulated by phosphorylation at a specific site that prevents guanine nucleotide conversion from the di- to the tri-phosphorylated state and thus impairs recycling of this translation initiation factor (Datta et al. 2003).

It has been postulated that adherence to rigid structures, e.g. microtubules, in the cell is important to the assembly of the protein translation complex. In bladder and breast cancer, the cytoskeletal framework (which utilizes keratins) has been demonstrated to provide support for this translation complex (Heuijerians et al. 1989). It is interesting to note that reduced expression of keratin 15 (KRT15) is associated with increased expression of the translation factor EIF2S1 in the prediction model for increased elastase, which in turn predicts a lower stage tumor. This rule can be used to construct a mechanism for reduced keratin production to reduce elastase protein synthesis in spite of increased transcription of elastase, and vice verse. Of note, higher levels of cytokeratin 15 are expressed in other forms of neoplasia (Jih et al. 1999).

The role for the mitochondrial ribosomal precursor is not clear, although decreased expression might imply a lesser rate of metabolism within the mitochondria. It is interesting to note that overexpression of MRPL19 does not associate with other molecular species to predict low levels of elastase, which in turn predicts a higher stage tumor.

### TRIP12 and NAPG

Rule # 4./Depth 3

(TRIP12 ≤ 1176) & (NAPG ≤ 243) → (MRPL19 ≤ 161.4)

[acc: 97.4% cov: 53/61]

Rule # 10./Depth 3

(TRIP12 > 1176) → (MRPL19 > 161.4)

[acc: 75.8% cov: 5/61]

Rule # 11./Depth 3

(TRIP12 ≤ 1176) & (NAPG > 243) → (MRPL19 > 161.4)

[acc: 70.7% cov: 3/61]

The thyroid receptor interacting protein 12 (TRIP) is a component of PA700, an ATP-dependent multi-subunit protein that activates proteolytic activities. It interacts with the ligand binding domain of the thyroid hormone receptor (in a thyroid hormone T3-independent manner) and with retinoid X receptor (RXR). The activator for the thyroid and retinoic acid receptor is over-expressed in approximately 60% of breast cancers and it has been reported to “cooperate” with her-2-neu, a breast oncogene associated with aggressive tumor, in dysregulation of transcription factor ER81 (Goel and Janknecht, 2004).

N-ethylmaleimide-sensitive factor attachment protein, gamma (NAPG) is required for vesicular transport between the endoplasmic reticulum and the Golgi apparatus. A relationship between a protein that interacts with a membrane bound receptor (i.e. TRIP12) and transport from the endoplasmic reticulum and the Golgi apparatus, and from there to the membrane surface, is plausible. It is not clear how this actually down-regulates expression of MRPL1. It is interesting to note that TRIP12 and MRPL19 are located on the same arm of chromosome 2 (see Table 2). It is possible that TRIP12 and MRPL19 share a regulatory mechanism based on the proximity of their genes on chromosome 2.

### FRDA

Rule # 5./Depth 3

(FRDA > 37.8) → (EIF2S1 > 52)

[acc: 97.6% cov: 57/61]

Frataxin in its mutated form causes the neurodegenerative disease Friedrich’s ataxia. Up-regulation of the mitochondrial precursor to frataxin (FRDA) predicts increased expression of EIF2S1. There have been scattered reports implying an association between frataxin and neoplastic disease. In one report, over-expression of frataxin was associated with resistance to cis-platinum in ovarian carcinoma cell lines (Ghazizadeh, 2003). This was attributed to increased de-toxification of therapy by frataxin. The patients in this study did not receive chemotherapy prior to surgery. FRDA only appears in the rules for low risk disease.

### CTRL and IDS

Rule # 6./Depth 3

(CTRL > 194.4) & (IDS ≤ 163.3) → (KRT15 <= 616.8)

[acc: 97.5% cov: 54/61]

Rule # 13./Depth 3

(CTRL ≤ 194.4) → (KRT15 > 616.8)

[acc: 70.7% cov: 4/61]

Rule # 14./Depth 3

(CTRL ≤ 194.4) & (IDS > 163.3) → (KRT15 > 616.8)

[acc: 45.3% cov: 3/61]

Chymotrypsin like protease precursor (CTRL) is associated with cancer cachexia (e.g. Wyke et al. 2004). There is no known association with iduronate 2-sulfatase precursor in cancer research. IDS is important in the degradation of heparan sulfates, which in turn are believed to inhibit proliferation in fibroblasts but not in tumor cells (Cheng et al. 2001). It is interesting to note that CTRL and IDS share a general function: catabolism. The glycose aminoglycans, of which heparin sulfate is one, are biopolymers with lubricant qualities. The related metabolic function of the two transcripts is intriguing and gives this association a plausible status.

### PLAB, H3FD, ANXA5 and DDX5

This rule that predicts up-regulation of alpha-1-antitrypsin is discovered in backward chaining of the high risk group. However, it is interesting to see that the coverage for the conditions for rule #15 at 44 of 61 patients is higher than the number of high risk patients (19) or the number of patients with Stage III lung cancer (13). This is, of course, explained by the fact that alpha-1-antitrypsin alone does not predict stage III or high risk disease.

Rule # 15./Depth 2

(PLAB ≤ 3703.9) & (H3FD ≤ 167.6) & (ANXA5 > 750) & (DDX5 < 2804.7) → (SERPINA 1 > 65)

[acc: 96.9% cov : 44/61]

This rule is interesting because nearly every one of its constituents has been associated with some sort of neoplastic behavior. Growth differentiation factor 15 (PLAB) is part of the TGF-β signaling family of proteins. It has been reported in association with several cancer types, including lung cancer (Kannan et al. 2000) and prostate cancer (Lindmark et al. 2004). In this case low levels of PLAB and member D of the H3 histone family (H3FD) combine with high levels of annexin V and an RNA-dependent helicase (p68) to result in up-regulation of alpha-1-antitrypsin. A high level of helix unwinding protein combined with a low level of histones would be most consistent in a cell with active transcription and replication.

The H3 histone family proteins are functionally associated with cancer, and more specifically lung cancer (Suh et al. 2002; Koesller et al. 2003; Tani et al. 2004). De-acetylation of histones leads to gene silencing by formation of nucleosomes. Inhibitors of histone deacetlyation (inhibitors of gene silencing) lead to apoptosis in some tumor cell lines (Zhu et al. 2001). The association of low transcript in rule #15 may either reflect high levels of H3 histone family, member D (H3FD) in response to high levels of de-acetylation, causing repression of transcription. Alternatively, if it truly reflects low levels of histone family 3, the higher transcription rates are also plausible in predicting higher levels of alpha-1-antitrypsin transcripts. The association of lower levels of helicase transcript (DDX5) with low levels of H3D transcript implies that overall the transcription rates could be decreased. Again, negative feedback on DDX5 from excessive helicase would result in low transcript levels and a paradoxical result of increased cellular transcription. Overexpression of some helicase genes has been observed with some sarcoma related cell lines (Bai et al. 2000). In a comparison of cell lines, RNA helicase A was shown to be more highly expressed in small cell lung cancer compared to non-small cell lung cancer, which may also be reflected in the relatively low transcription level in rule #15 (Wei et al. 2004). In a separate study focused on p68 (DDX5), colon cancer cell lines with decreased p68 expression were less well differentiated (Singh et al. 1995). This data would also support the extended lineage of association of increased alpha-1-antitrypsin expression with high risk disease.

Annexin V has been studied in the context of apoptosis (Ravassa et al. 2004). A key step in lung tumor progression is escape from apoptosis. Annexin V expression is associated with apoptosis secondary to therapy in small cell lung cancer cell lines (Bjorkhem-Bergman et al. 2004). Again, in this case the higher level of transcript may reflect a low level of protein, and therefore resistance to apoptosis. The association of annexin V and histones in rule #15 is also interesting because of the observed increase in apoptosis secondary to inhibition of gene silencing by histone (Zhu et al. 2001).

This rule has been assigned a score of 2 for interesting associations because of the related functions of histones, helicase, and growth differentiation factors in modulating transcription.

### POM121 and PLAB

Rule # 16./Depth 3

(KIAA0618 > 27.2) → (PLAB ≤ 3703.9)

[acc: 95.5% Data cov: 54/61]

While there are no reports of POM121 specifically associated with lung cancer or a neoplastic process of any kind, there are reports of increased expression of nuclear pore proteins in some cancer (e.g. Slape and Aplan, 2004).

### SC4MOL and H3D

Rule # 17./Depth 3

(SC4MOL > 32) → (H3FD ≤ 167.6)

[acc: 97.5% cov: 54/61]

The sterol methyl oxidase-like gene SC4MOL does not have any specific references in connection with lung cancer or neoplasia in general. However, steroid metabolism is important considering the effect of steroid signals associated with neoplasia (e.g. estrogen, progesterone). The up-regulation of a steroid predicting the down regulation of histones could reflect a change in transcription activity.

### AKAP13, SCL14A2 and ANXA5

Rule # 18./Depth 3

(AKAP > 496) & (SLC14A2 ≤ 397.1) → (ANXA5 >750)

[acc: 97.4% cov: 53/61]

There are several publications relating the A-kinase anchoring protein AKAP13 and cancer (e.g. Sterpetti et al. 1999). It is in fact very interesting that a signal transduction molecule (AKAP13) predicts phospholipase transcription as phospholipases also function in signaling. The role of a urea transport protein likely reflects underlying amino acid use. As an example, arginine has numerous roles in cellular metabolism, including the urea cycle, nitric oxide synthesis, and cell growth and healing processes, all important in functions that promote cancer (Lind, 2004).

### KRT13 and DDX5

Rule # 19./Depth 3

(KRT13 ≤ 262.9) → (DDX5 ≤ 2804.7)

[acc: 97.6% cov: 57/61]

It has been postulated that proximity in gene loci for two or more genes in instances of similar expression patterns suggests related transcription control mechanisms. A change in the expression pattern of one gene then predicts the same direction of change in the other. Embedded in the rules that predict Stage 3 disease is a rule that predicts DDX5 to be below some threshold value when KRT13 is below some threshold value.

KRT13 is keratin 13, a cytoskeletal protein that functions in maintaining the integrity of the cell shape and may also function as a support in translation; DDX5 is a DNA unwinding protein that functions in DNA replication. KRT13 is located at 17q12 to 17q21.2, while DDX is located at 17q21. This suggests that the transcription of DDX5 is associated with transcription of KRT13. One could further postulate that the two are controlled together because the skeletal framework is expanded when a cell is preparing to undergo mitosis (cell division), which also requires DNA replication. Higher mitotic rates would lead to a larger tumor mass, and therefore a Stage III tumor. Interestingly, Massion and Carbone (2003) describe amplifications (increased numbers of copies of genes) in the 17q region of the genome (chromosome 17) associated with non-small cell lung cancer. This discovery using C45W-BCRI supports that association.

### Further Steps Towards (Semi-) Automating Hypothesis Generation on Gene Pathways

We have illustrated in the previous section how induced rules and literature review combine to create hypotheses about gene interactions. Our longer term goals are to (semi-)automate this time-consuming process by interfacing BCRI rule discovery with prior knowledge in the form of pathway software tools.

We will focus here on C45W-BCRI rules that are reflected in pathways based on existing knowledge. We used both Metacore (Nikolsky et al. 2005) Pathway Assist™ (Nikitin et al. 2003) to build the shortest pathways using gene expression attributes of the rules induced with C45W-BCRI as nodes. Using Pathway Assist™, which provides machine readable output, we identified nodes in the pathways where we can automate hypothesis generation using this combination of induced and existing knowledge. We start here with an example in which there is evidence for a rule induced from C45W-BCRI using Pathway Assist™.

*Example 1*: In our C45W-BCRI Rule 8./1, we have

8./1(ELA2 ≤ 163.3) & (SERPINA1 > 65) → (Stage = 3) [acc: 89.1% cov: 12/61]

**Figure 3.**
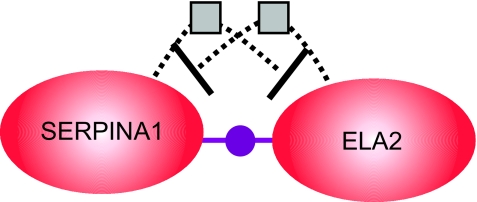
Pathway Assist™ diagram showing SERPINA1 and ELA2 relationships of Example 1. The protein products are indicated by the large ovals, a binding interaction is indicated by the purple dot relationship between the ovals, and gene expression regulation is indicated by a square along a dotted line.

From C45W-BCRI we have induced that ELA2 and SERPINA1 interact in a negative fashion to predict a Stage 3 presentation. Pathway Assist™ shows that ELA2 and SERPINA1 are tightly coupled nodes in which the protein products of these genes both bind together and also act to down-regulate one another’s gene expression (see Figure 3). Table 6 summarizes the pathway data in a machine readable format.

Our point here is that a semi-automated examination of the pathway relationship for the attributes selected by BCRI would have identified this rule as consistent with existing information. We present this example as a template upon which to build an automated conjunction of the BCRI output with a structured database such as Pathway Assist. The identification of the actual nature of the relationship provides more information than simply an ontology look-up for each of the genes, which has been one of the preferred methods of inferring pathway information as a part of the analysis of gene expression array data. Once an interaction is identified as already existing in the pathway database, it can be automatically selected as a plausible hypothesis, that is that the interaction of these two genes plays a role in the outcome of interest.

*Example* 2: As a second example, consider Rule 5./3,

5./3 (FXN > 37.8) → (EIF2S1 > 52)

[acc: 97.6% cov: 57/61]

**Figure 4.**
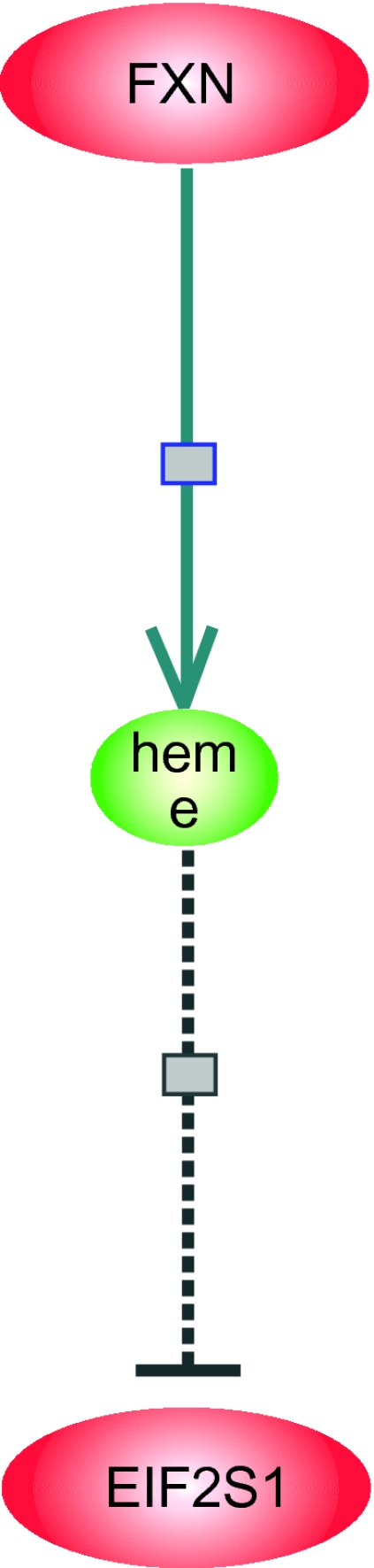
Pathway Assist™ diagram illustrating FXN and EIF2S1 relationships of Example 2

Pathway Assist™ shows that FXN has an “unknown” effect on the molecular synthesis of heme, the interaction represented as a solid line with a square in Figure 4, and that heme, a small molecule depicted by the small, central oval, inhibits the gene expression of EIFS2. The interaction is summarized in Table 7.

From our C45W-BCRI rule, if we accept that elevated gene expression of FXN leads to elevated levels of its protein product frataxin, then we can *infer* that frataxin *blocks* the molecular synthesis of heme to results in elevated expression of EIFS2. Thus, the inductively derived rule, which might be tentatively abstracted as (FXN → EIF2S1, effect positive), together with (heme—|EIF2S1, effect negative) from prior knowledge, suggests that (FXN→ heme, effect *negative*, in place of unknown). This example illustrates where *induction can suggest fillers for gaps in background knowledge.*

*Example 3:* In Rule 19./3, 23 have from C45W-BCRI

19./3 (KRT13 ≤ 262.9) → (DDX5 ≤ 2804.7)

**Figure 5.**
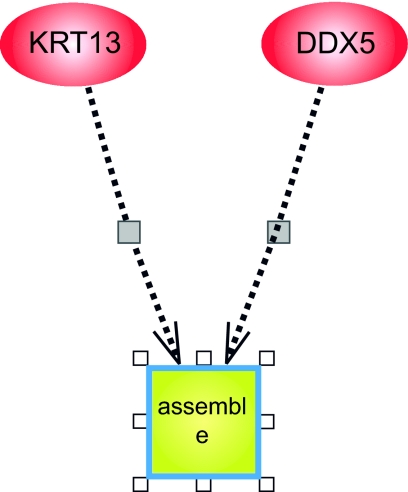
Pathway Assist™ diagram of KRT13 and DDX5 relationships of Example 3

[acc: 97.6% cov: 57/61]

and from Pathway Assist™ we have the diagram of Figure 5 and description of interactions in Table 8. The square labeled “assemble” represents a cell function of assembly, for example assembling a scaffold of filamentous proteins either into a structure for cell shape, or a scaffold upon which catalyzed reactions can take place.

We have already discussed that this rule is interesting from the perspective of the proximity of these two genes on chromosome 17. Taking a different approach and using Pathway Assist™, we see that both the protein product of KRT13 and of DDX5 have an unknown role in assembly. From the BCRI rule we see that low expression of KRT13 predicts low expression of DDX5. Thus, we can hypothesize that there is a more tightly coupled relationship in the effect of KRT13 and DDX5 on assembly.

## Summary

Knowledge discovery from data includes hypothesis generation and hypothesis testing. There is a paucity of formal, (semi-) automated methods for hypothesis generation about gene interactions (e.g. Quackenbush, 2005). Gene expression microarray data, where hindered by the sparse number of samples, may lend itself better to hypothesis generation than prediction model building (e.g. Hoos and Cordon-Cardo, 2001; Anzick and Trent, 2002). We have investigated backward chaining rule induction as a strategy for limiting the search for associations to a manageable set of relationships to postulate governing gene networks in the context of lung cancer survival.

BCRI can be applied in other domains as well, by initiating the process with different top level goals/classes. Regardless of the context, the goal of BCRI is to limit hypothesis generation to a manageable set of relationships for expert scrutiny. Experts can then assess the plausibility of rules (uncovering mechanisms) and the utility of rules (discovering clinical applications).

Thus far, this is an exploratory study of one implementation of the BCRI paradigm. An evaluation of the rules discovered suggests that conditioning the space of associations that is searched on some meaningful, overriding task/classification may better yield a rule set that is densely-populated with mechanistically plausible rules. Our prototype implementation of BCRI is not optimal from a time-cost standpoint. Furthermore, our current implementation using C4.5 appears to rarely find what amounts to OR-nodes in a rule network. A non-greedy system such as Brute (Riddle et al. 1994), could be used as ***RuleInducer***, which would more liberally introduce OR nodes into the rule base, and thus would correspond to alternative, hypothesized pathways. Future work will look at other rule discovery systems as the core of BCRI, as well as more tightly couple the wrapper and core method to improve efficiency.

Our evaluation strategy compares BCRI-induced interactions against prior knowledge. Future work will focus on exploiting prior knowledge (e.g. signal transduction and regulatory networks). We plan to map BCRI induced interactions onto known composites of signaling and regulatory networks, as described above in the Discussion but in a more (semi-)automated fashion.

We can also use this mapping to bias induction (Evans and Fisher, 2002; Ortega and Fisher, 1995) and supplement or revise, as needed, existing knowledge from induced knowledge (Mooney, 1993) in a “systems biology” approach. To this effect, we have recently completed work using an iterative approach that uses the networks learned from BCRI combined with existing knowledge to discover modifiers to known signaling networks that qualify their relevance to outcome in lung cancer patients (Frey et al. 2005). This approach could be especially effective in understanding the effectiveness of molecularly targeted therapies for lung cancer.

Finally, we will evaluate BCRI using other criteria. We realize that BCRI’s search through rule space will miss many associations. However, our goal is not to discover all the plausible rules that govern gene interactions, but to reduce them to a manageable number that is enriched for relevance to survival. Thus, we expect to have a high density of relevant rules using BCRI. One relevant comparison is against unsupervised association rule learners (Mannila, 2002) in terms of the number of rules learned, and the density of “interesting” rules, though this latter criterion may be difficult to formalize in a comparative study. We are also interested in comparing prediction accuracy of the rule network learned by BCRI against standard rule induction engines. To exploit the inference procedures of rule-based expert systems will require that we modify BCRI to produce rules that incorporate uncertainty (e.g. variance in antecedent thresholds, variance around accuracy point estimates of rules). Recent work by Waitman et al. (2003) provides guidance on how this can be done. Waitman et al (2006) also show how similarity between rules can be computed, and rules can be visualized in terms of this similarity metric using multidimensional scaling. The identification of clusters in this visualization may provide additional information to filter rules for expert scrutiny.

In summary, the inference possibilities of a rule network constructed through backward-chaining rule induction are intriguing. ***We emphasize that it is not our goal in this paper to evaluate the accuracy of BCRI-induced rule networks for predicting clinical outcome.*** As described in the Introduction to this paper, we address here the paradigm of discovering gene interactions that are operational within a survival class, and therefore are relevant to therapies targeted for that class. Our primary goal is a limited, focused exploration of the associations between variables. Hypothesis generation in high density data with an effectively infinite number of combinations to examine requires an automated, computer tool for searching the plethora of possible interactions, and presenting selected possibilities (e.g. selected by heuristics involving accuracy and coverage, and heuristics relating to top-level outcomes of interest) to an expert analyst for comment and scrutiny.

As we stated at the start of this section, gene expression microarray data may lend itself better to hypothesis generation than prediction model building (e.g. Hoos and Cordon-Cardo, 2001; Anzick and Trent, 2002). BCRI as a data mining strategy has been developed in response to this paradigm, and not as a classifier method. Validation of BCRI results comes from previous knowledge in the published literature and curated pathways databases, or from future bench level research. BCRI is not a tool for building a classifier and therefore is not validated by testing its outcome on a separate test set. An interaction that has been previously described in the context of lung cancer survival can be considered as validated, if that knowledge support does exist. In most cases, the literature support is partial, and in other cases not existent at all. Thus, there is an ordering of the hypotheses generated from BCRI from those fully validated through partially validated to completely novel. With BCRI, we achieve our goal to devise a computer strategy that can explore a very large space of gene interactions, enriched for plausibility, and reduce them to a manageable set for human consideration and subsequent study.

## Figures and Tables

**Table 1.** **Pseudocode for BCRI.** Data is a data set such as the Beer et al. data set. Classes is a set of class labels that are included in and used to classify Data. RuleInducer is a function of two parameters (i.e. a supervised rule induction system) that learns if-then rules to predict a TargetCond from a DataSet. PriorityFn is a function that takes an if-then rule a returns a floating point priority value associated with the rule. This priority value is used to order the rule on a priority queue, and this priority queue is used to guide the exploration of rules to which backward chaining is applied. TerminateFn is a function that decides whether a given rule should be backward chained.

Function Wrapper-BCRI
Returns a RuleSet
With parameters DataSet Data
TargetSet Classes
RuleSet Function RuleInducer (DataSet, TargetCond)
float Function PriorityFn (Rule),
bool Function TerminateFn (Rule) {
PQ = InitializePriorityQueue(PriorityFn);
FOR each class in Classes, Enqueue(PQ, [class **→**___ ]);
WHILE (NOT Empty(PQ)) {
R = Dequeue(PQ);/* and place R in Results SET*/
IF (NOT TerminateFn(R) {
FOR each a IN ANTECEDENTS(R) {
Children = RuleInducer (Data, a);
FOR each c IN Children Enqueue(PQ, c)
}
}
}/* end WHILE */
}/* end BCRI */

**Table 2.** **Gene Names, Locus, Function, and Rule Depth.** The genes are grouped according to their gene locus. Rule depth, described in the text, is given to indicate the closeness of the terms in the rules. Note that many genes appear at the same rule depth for both high and low risk classes. Gene transcripts located on the same chromosome arm are shown in bold if their rules are within a single depth unit of one another, suggestive of related transcription control. Abbreviations are HUGO compliant except where noted by an asterisk. Locus is based on the LocusLink information and Function is based on the Gene Ontology information as reported in Genecards.

**Gene Symbol**	**Name**	**Locus**	**Function**	**Rule Depth for**	
				Low Risk	High Risk
IDS	Iduronate 2-sulfatase precursor	Xq28	metabolism	3	3
MRPL19	60S ribosomal protein L19, mitochondrial precursor	2q11.1–11.2	protein biosynthesis	2	2
TRIP12	Thyroid receptor interacting protein 12	2q36.3	ubiquitin-protein ligase activity	3	3
ANXA5	Annexin V	4q28–q32	phospholipase inhibitor activity		2
SC4MOL	Sterol C-4 methyl oxidase-like	4q32–q34	steroid metabolism		3
H3FD *(HIST1H3E)	H3 histone family, member D (H3FD)	6p21.3	chromosome organization		2
KIAA01618* (POM121)	Nuclear envelope pore membrane protein (POM121)	7q11.23	transport		3
FRDA	Frataxin, mitochondrial precursor	9q13–q21.1	inositol/phosphatidylinositol kinase activity	3	
EIF2S1	Eukaryotic translation initiation factor 2 subunit 1	14q23.3	protein biosynthesis	3	
SERPINA1	Alpha-1-antitrypsin precursor	14q32.1			1
AKAP13* (LBC)	A-kinase anchoring protein	15q24–25	intracellular signaling cascade		3
CTRL	Chymotrypsin-like protease CTRL-1 precursor	16q22.1	proteolysis and peptidolysis	3	3
KRT13	Keratin, type I cytoskeletal 13	17q12–q21.2	structural constituent of cytoskeleton	3	
DDX5	Probable RNA-dependent helicase p68	17q21	ATP-dependent helicase activity		2
KRT15	Keratin, type I cytoskeletal 15	17q21.2	structural constituent of cytoskeleton		3
NAPG	N-ethylmaleimide-sensitive factor attachment protein, beta	18p11.21	Intracellular transporter activity	3	3
SLC14A2	Urea transporter, kidney	18q12.1–q21.1	urea transport		3
ELA2	Leukocyte elastase precursor	19p13.3	proteolysis and peptidolysis	1	1
PLAB (GDF15)	*Growth differentiation factor 15	19p31.1–13.2	signal transduction (TGF-β)		2

**Table 3.** **Rules induced by backward chaining of Risk top-level categories.** The ordering of rules is not strictly indicative of the order in which they were discovered. Rule number is given with its associated depth in the backward chaining process. Indentation indicates a parent child relationship. “acc” denotes accuracy of prediction and “cov” denotes the number of cases covered by the IF condition over the total number of samples. A notation of *(i)* indicates that this rule is interesting either because of an established association in the literature or because of a plausible hypotheses that can be inferred from the rule.

	**(Risk = Low)→**	
Rule #/Depth		
1./0	(Stage = 1) → (Risk = Low)	
2./1	(ELA2 > 163.3) → (Stage = 1) [acc: 94.4% cov: 46/61]	*(i)*
3./2	(MRPL19 ≤ 161.4) & (EIF2S1 > 52) & (KRT15 ≤ 616.8) → (ELA2 > 163.3) [acc: 97.0% cov: 45/61]	*(i)*
4./3	(TRIP12 ≤ 1176) & (NAPG ≤= 243) → (MRPL19 ≤ 161.4) [acc: 97.4% cov: 53/61]	*(i)*
5./3	(FRDA > 37.8) → (EIF2S1 >52) [acc: 97.6% cov: 57/61]	
6./3	(CTRL > 194.4) & (IDS ≤ 163.3) → (KRT15 ≤ = 616.8) [acc: 97.5% cov: 54/61]	*(i)*

	**(Risk = High) →**	
7./0	(Stage = 3) → (Risk = High)	
8./1	(ELA2 ≤ 163.3) & (SERPINA1 > 65) → (Stage = 3) [acc: 89.1% cov: 12/61]	*(i)*
9./2	(MRPL19 > 161.4) → (ELA2 ≤ 163.3) [acc: 84.1% cov: 8/61]	
10./3	(TRIP12 > 1176) → (MRPL19 > 161.4) [acc: 75.8% cov: 5/61]	*(i)*
11./3	(TRIP12 ≤ 1176) & (NAPG >= 243) → (MRPL19 > 161.4) [acc: 70.7% cov: 3/61]	
12./2	(MRPL19 ≤ 161.4) & (KRT15 > 616.8) → (ELA2 ≤ 163.3) [acc: 79.4% cov: 5/61]	*(i)*
13./3	(CTRL ≤ 194.4) → (KRT15 > 616.8) [acc: 70.7% cov: 4/61]	
14./3	(CTRL > 194.4) & (IDS > 163.3) → (KRT15 > 616.8) [acc: 45.3% cov: 3/61]	
15./2	(PLAB ≤ 3703.9) & (H3FD ≤ 167.6) & (ANXA5 > 750) → (DDX5 ≤ 2804.7) → (SERPINA1 > 65) [acc: 96.9% cov: 44/61]	*(i)*
16./3	(KIAA0618 > 27.2) → (PLAB ≤ 3703.9) [acc: 95.5% Data cov: 57/61]	*(i)*
17./3	(SC4MOL > 32) → (H3FD ≤ 167.6) [acc: 97.5% cov: 54/61]	
18./3	(AKAP13/LBC > 496) & (SLC14A2 ≤ 397.1) → (ANXA5 > 750) [acc: 97.4% cov: 53/61]	*(i)*
19./3	(KRT13 ≤ 262.9) → (DDX5 ≤ 2804.7) [acc: 97.6% cov: 57/61]	*(i)*

**Table 4.** **Density of References for Molecular Species (Gene or Gene Product) in Rules**. To be considered a positive finding (+), a reference linking the gene specified in the row with either lung cancer (column 2) or with cancer in general (column 3) must have been identified in a PubMed search. Otherwise, there is no known correlation (–).

**Gene Name**	**Lung Cancer References**	**Other Cancer References**
ELA2	+	+
SERPINA1	+	+
MRPL19	−	−
EIF2S1	+	+
KRT15	−	+
TRIP12	−	+
NAPG	−	−
FRDA	−	+
CTRL	−	+
IDS	−	−
ANXA5	+	+
PLAB*(GDF15)	+	+
H3FD*(HIST1H3E)	+	+
DDX5	−	+
AKAP13	+	+
SLC14A2	−	−
KIAA01618* (POM121)	−	+
SC4MOL	−	+
KRT13	−	−
19	6	12

**Table 5.** **Knowledge Introduced by Rule**. The integer 1 is used to indicate a positive result in response to the question presented at the head of the column and 0 is used to indicate a null result.

Rule #	Corresponds to to a previously established or hypothesized interaction for lung cancer or cancer in general?	Supports, specializes, or contradicts a previously forwarded interaction?	Suggests a new question?	Has a plausible hypothesis for a mechanism requiring further study?	Coverage
2	1	1	0	1	46/61
3	1	1	0	1	45/61
4	0	0	1	1	53/61
5	0	0	1	0	57/61
6	0	0	1	1	54/61
8	1	1	0	1	12/61
9	0	0	1	0	8/61
10	0	0	1	1	5/61
11	0	0	0	0	3/61
12	1	1	0	1	5/61
13	0	0	1	0	4/61
14	0	0	1	0	3/61
15	1	1	1	2	44/61
16	0	0	1	1	57/61
17	0	0	1	0	54/61
18	0	0	1	1	53/61
19	0	0	1	1	57/61

17Rules	5 Previously Hypothesized	5 Specialization of Hypothesis	12 New Associations	12 Plausible Associations	

**Table 6.** Details of Example 1 relationships given by Pathway Assist™.

**Type**	**Nodes**	**Effect**
Binding	ELA2 ---- SERPINA1	
Regulation	ELA2 ---| SERPINA1	negative
Regulation	SERPINA1 ---| ELA2	negative

**Table 7.** Details of Example 2 relationships given by Pathway Assist™.

**Type**	**Nodes**	**Effect**
Regulation	heme ---| EIF2S1	negative
MolSynthesis	FXN ---> heme	unknown

**Table 8.** Details of relationships of Example 3 given by Pathway Assist™.

**Type**	**Nodes**	**Effect**
Regulation	KRT13 ---> assemble	unknown
Regulation	DDX5 ---> assemble	unknown

**Figure 6.**
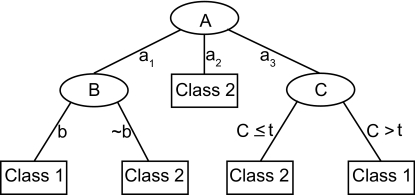
A decision tree.

**Figure 7.**
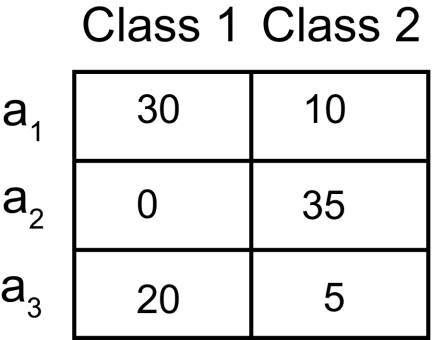
Joint distribution of attribute A and Class

**Figure 8.**
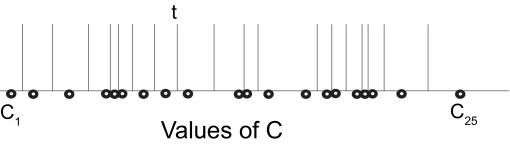
Possible threshold values for a continuous attribute, C

## Footnotes

1 A rule induction engine of the type that we will describe in the Methods Section and Appendix A learned the example rule above, including the choiceof thresholds for continuous attributes.

## Appendix A

### Adaptation of C4.5 for BCRI

C4.5 is a well-known machine learning system for building a classifier of if-then rules. An adaptation of C4.5 was used in the role of ***RuleInducer*** for our implementation of BCRI.

In particular, C4.5 builds a decision tree, illustrated in Figure 6, where each path of the decision tree corresponds to an if-then rule. For example, the rightmost path corresponds to the rule: IF (A = a_3_) AND (C > t) THEN Class = 1. A decision tree is a *classifier*, meaning that every possible datum is classified by (matches) one and only one path/rule. At the end of this section we will discuss how a C4.5-generated decision tree is adapted to the specification of ***RuleInducer***, but for now we focus on how C4.5 builds a decision tree from data.

#### C4.5 Overview

C4.5 is a recursive, greedy algorithm for building a decision tree from the top down. Starting with the empty decision tree and the set of training data, C4.5 must first select the attribute that will be tested at the root of the final decision tree. It selects that attribute with values that “best” predicts class with respect to the training data. For example, let’s assume that there are 100 training examples that were used to construct the tree of Figure 6. Further assume that these 100 examples are distributed jointly with respect to attribute A and Class as shown in Figure 7. Figure 7 shows, for example, that 40 examples total have value a_1_ for attribute A, and of these 40 examples, 30 are members of class 1 and 10 are members of class 2. Each value of attribute A predicts one class over another with varying degrees of certainty as assessed using the training data.

In C4.5 the measure of “best” is based on an information-theoretic measure of entropy, which is minimized ideally. We will not detail this measure here, but we appeal to intuition that attribute A scores well (small) in terms of entropy: each value of attribute A is associated with a class that dominates to varying degrees. In the very best case, entropy is minimal when each and every value of attribute is associated with members of one class only (e.g. as value a_2_ is in Figure 7). In the worst case, entropy is maximal when each and every attribute is associated with equal numbers of members from each possible class (i.e. the attribute has no predictive value).

In selecting the root attribute, the entropy of all attributes are measured and the one that minimizes C4.5’s entropy-based measure is selected. In our example, this is attribute A. Since attribute A has three values, the training data is partitioned by these three different values, and for each subset, a recursive call is made to C4.5. For example in the leftmost branch, over the subset of data having A=a_1_, B is selected as the attribute that best predicts class *within this subset*. Note that B is a binary-valued attribute, which is a special case of a nominal attribute (e.g. A), and entropy is computed in the same manner as with any other nominal attribute.

#### Assessing continuous attributes

Assessing continuous attributes requires special consideration in C4.5. In our example of Figure 6 we show that attribute C is selected as the best attribute for the training data subset corresponding to A = a_3_. Attribute C is continuous and C4.5 selects a threshold, t, to divide the values of C. In particular, C4.5 sorts the values of C, and considers each median between two consecutive values as a possible threshold as illustrated in Figure 8.

For each possible threshold the entropy is computed, and the threshold with the minimal entropy is selected. The entropy for this best threshold then serves as the entropy of the attribute, and is used in comparisons against the entropies of other candidate attributes. In our example of Figure 6, presumably attribute C thresholded at t had smaller entropy than any other attribute.

#### Terminating recursion

On each recursive call to C4.5, the best attribute for an increasingly small training data subset is selected until one or more termination conditions is satisfied. One termination condition is if all training data are members of the same class, as is the case with a_2_. Another termination condition is if the data subset currently being considered is less than a specified number (e.g. 5).

When a termination condition is satisfied, a leaf in the decision tree is created, which gives the class that dominates in the current subset of training data.

#### C4.5 Miscellany

C4.5 builds a decision tree as described above. It has a number of parameters available. In all cases we used default settings for these. In the option of the C4.5 package that we use, C4.5-rules, each path of the decision tree is then translated straightforwardly into an if-then rule. There can be some additional massaging of these rules (e.g., a “pruning” operation), but we do not detail that process here.

Importantly, the set of if-then rules that results contains rules with consequents across the possible classes. That is, there will be rules that predict both Class 1 and rules that predict Class 2 is the example of Figure 6. To satisfy the specification of ***RuleInducer***, only rules predicting *TargetCondition* are retained. The nonoptimality of the wrapper design that we are using currently stems from the need to call C4.5 multiple times, once to get the rules for Class 1 and once to get the rules for Class 2, even though these rules are both obtainable on one call. Again, the wrapper design choice stems from a desire to easily substitute in other rule induction systems, such as Brute, which learn rules specific to a *TargetCondition* and no others.
